# IL-10 Conditioning of Human Skin Affects the Distribution of Migratory Dendritic Cell Subsets and Functional T Cell Differentiation

**DOI:** 10.1371/journal.pone.0070237

**Published:** 2013-07-18

**Authors:** Jelle J. Lindenberg, Dinja Oosterhoff, Claudia C. Sombroek, Sinéad M. Lougheed, Erik Hooijberg, Anita G. M. Stam, Saskia J. A. M. Santegoets, Henk J. Tijssen, Jan Buter, Herbert M. Pinedo, Alfons J. M. van den Eertwegh, Rik J. Scheper, Hans J. P. M. Koenen, Rieneke van de Ven, Tanja D. de Gruijl

**Affiliations:** 1 Department of Medical Oncology, VU University medical center, Amsterdam, The Netherlands; 2 Department of Pathology, VU University medical center, Amsterdam, The Netherlands; 3 Laboratory of Medical Immunology, Department of Laboratory Medicine, Radboud University Nijmegen Medical Centre, Nijmegen, The Netherlands; Istituto Superiore di Sanità, Italy

## Abstract

In cancer patients pervasive systemic suppression of Dendritic Cell (DC) differentiation and maturation can hinder vaccination efficacy. In this study we have extensively characterized migratory DC subsets from human skin and studied how their migration and T cell-stimulatory abilities were affected by conditioning of the dermal microenvironment through cancer-related suppressive cytokines. To assess effects in the context of a complex tissue structure, we made use of a near-physiological skin explant model. By 4-color flow cytometry, we identified migrated Langerhans Cells (LC) and five dermis-derived DC populations in differential states of maturation. From a panel of known tumor-associated suppressive cytokines, IL-10 showed a unique ability to induce predominant migration of an immature CD14^+^CD141^+^DC-SIGN^+^ DC subset with low levels of co-stimulatory molecules, up-regulated expression of the co-inhibitory molecule PD-L1 and the M2-associated macrophage marker CD163. A similarly immature subset composition was observed for DC migrating from explants taken from skin overlying breast tumors. Whereas predominant migration of mature CD1a^+^ subsets was associated with release of IL-12p70, efficient Th cell expansion with a Th1 profile, and expansion of functional MART-1-specific CD8^+^ T cells, migration of immature CD14^+^ DDC was accompanied by increased release of IL-10, poor expansion of CD4^+^ and CD8^+^ T cells, and skewing of Th responses to favor coordinated FoxP3 and IL-10 expression and regulatory T cell differentiation and outgrowth. Thus, high levels of IL-10 impact the composition of skin-emigrated DC subsets and appear to favor migration of M2-like immature DC with functional qualities conducive to T cell tolerance.

## Introduction

Dendritic cells (DC) are the major class of antigen presenting cells (APCs) regulating adaptive immunity. In the steady state, migratory immature DC from peripheral tissues take up antigen but lack the capacity to promote functional T cell-mediated immune responses. In response to activation signals, DC migrate to draining Lymph Nodes (LNs) and mature into potent immune-stimulatory APC that can drive T cell expansion and differentiation [1], [2]. As it is lined by a dense network of DC with ready access to lymph vessels, skin is a preferred site for the delivery of tumor vaccines [3]. Unfortunately, tumors exert powerful systemic immune suppression, resulting in impaired differentiation and activation of DC, possibly also impacting DC functionality in the skin.

Skin DC consist of two major migratory subsets: epidermis-derived Langerhans Cells (LC) and Dermal DC (DDC). The latter can be roughly divided into CD1a^+^ and CD14^+^ subsets [4]. All these subsets migrate to draining LN, even in the steady state, and so maintain peripheral tolerance [4]. Upon activation their migration rate increases and they acquire a mature phenotype, allowing the activation and expansion of specific T cells [4], [5]. LC have been suggested to preferentially activate Cytotoxic T Lymphocytes (CTL), whereas CD14^+^ DDC have been implicated in the priming of B cell responses [6]. However, these assertions are mostly based on findings from murine studies or from *in vitro* studies with LC or CD14^+^ DDC-like cells derived from CD34^+^ precursor cells. Functional studies with primary DC from human skin are complicated by their low numbers and therefore sparse. Nevertheless, some valuable information about the ability of human skin-associated DC subsets to direct T cell responses has been obtained.

Peiser *et al.* showed low-level release of IL-12p70 by LC, freshly isolated from epidermal sheets, as compared to monocyte-derived DC (MoDC) [7]. In line with this observation, others have suggested that the LC's supposed superior CTL activating ability might derive from its release of IL-15 or CD70 expression rather than IL-12p70 secretion [8]–[10]. One study demonstrated a superior ability of LC over DDC to induce both Th1 and Th2 responses [11], whereas others have shown primary human LC to skew Th cell responses (including conventional variant αβ-T cells restricted through CD1a) to a Th22-type response, characterized by IL-22 release in the absence of IL-17 [12], [13]. The T cell skewing abilities of DDC appear to be determined by a balance of factors in the microenvironment. Larregina and co-workers have shown in a number of reports that human DDC can skew Th cells to either a Th1, a Th2, or a Th17 profile, depending on their conditioning, number and activation state [14]–[16]. In the steady state LC are primarily responsible for the homeostatic proliferation of skin-resident memory T cells, most notably CD4^+^ regulatory T cells (Tregs) [17]. Recently, CD14^+^ DDC from human skin have been shown to induce Tregs [18]. As yet, it is unclear how tumor-mediated conditioning of the skin will impact the ability of the DC located there to skew and direct T cell responses. In this regard, modulation of the cutaneous cytokine balance may prove crucial to the efficacy of intradermally (i.d.) or epicutaneously applied cancer vaccines.

T cell activation by mature DC is a carefully coordinated process, involving a regulated sequence of events leading to DC migration, activation, cytokine release, and antigen presentation. For optimal immune activation to occur, these events need to be tightly controlled, both spatially and chronologically. In view of these considerations, we employed an organotypic human skin explant culture system to characterize migratory DC subsets and to study cytokine-related modulation of their phenotype and function and how this impacts CD4^+^ and CD8^+^ T cell activation and differentiation. Our results point to IL-10 as a major candidate effector of tumor-related modulation of DC subset migration from human skin, resulting in prevailing migration of CD14^+^BDCA3/CD141^+^ DC that acquire an immunoregulatory phenotype and cytokine release profile with detrimental effects on cell-mediated immunity.

## Materials and Methods

### Skin explant preparation and culture

Healthy human skin specimens were obtained after verbal informed consent at the time of hospital admission from patients undergoing corrective breast or abdominal plastic surgery at the VU University medical center (VUmc, Amsterdam, The Netherlands) or at the Tergooi hospital (Hilversum, The Netherlands) and used in an anonymous fashion. As this does not entail additional interventional procedures and applies to biological materials that would otherwise be disposed of, verbal consent sufficed according to Dutch national guidelines; patients that objected to this procedure signed a statement to this effect, in accordance with the “Code for Proper Use of Human Tissues” as formulated by the Dutch Federation of Medical Scientific Organizations (www.fmwv.nl) and following procedures approved by the IRB [19]. Materials from patients with breast cancer participating in clinical trials were collected after written informed consent according to protocols approved by the VUmc IRB (IRB00002991; IORG number 0002436).

Cytokines were i.d. injected in surgical healthy skin specimens with a Micro-Fine Insulin syringe (0.33 mm (29 G)×12.7 mm needle, BD, Franklin Lakes, NJ) in a total volume of 20 µl serum-free medium. The following amounts of cytokines were injected per explant: 100 ng GM-CSF (Berlex Laboratories Inc. Montville, NJ), 1000 IU IL-4, and 10 ng IL-10 (Strathmann Biotec, Hamburg, Germany). These amounts were based either on optimal effects or on maximal sub-toxic doses found upon titration [19]. At the site of injection a 5 mm-diameter urtica appeared and an exact punch biopsy of 6 mm was taken. The biopsy was lifted from the specimen with a sterile forceps and with sterile scissors the dermis was cut at a depth of 2–3 mm to obtain skin explants. Biopsies were placed in 1 ml culture medium (i.e. IMDM containing 5% Human Pooled Serum (Sanquin Blood Supply, Amsterdam, The Netherlands), supplemented with 100 IU/ml sodium penicillin (Yamanouchi Pharma), 100 µg/ml streptomycin sulfate (Radiumfarma-Fisiopharma), 2 mM L-glutamine (Invitrogen Life Technologies), and 0.01 mM 2-ME (Merck). Per experimental condition 10–20 explants were cultured. After a culture period of 48 h the skin explants were discarded. Migrated cells were harvested at these or later time points and used for further analyses –no additional cytokines were added to the cultures. Between 1998 and 2002, skin from mastectomy material was excised by an experienced pathologist from patients with breast cancer at the VUmc in Amsterdam, The Netherlands [20], [21]. Skin was i.d. injected with 20 µl serum-free medium. Explants were taken and cultured as described above, after which migrated cells were harvested and analyzed by flow cytometry. From four patients with Locally Advanced Breast Cancer (LABC, i.e. stage IIB with a primary breast tumor larger then 5 cm, IIIA or IIIB according to AJCC criteria), skin was collected and cultured subsequent to neoadjuvant chemotherapy with doxorubicin and cyclophosphamide [20], [21]. Partial tumor responses according to RECIST criteria were observed in all four patients; in three patients only microscopic evidence of disease remained whereas in one patient the tumor shrunk from a volume of 23×17 cm to a diameter of 1.5 cm.

### HLA-A2 typing of the skin explants

Medium-injected 6 mm biopsies (10 per condition) were placed in 10 cm diameter culture dishes containing 15 ml 0.05% trypsin (Invitrogen GIBCO, Paisley, Scotland) for 4–5 hr at 37°C, 5% CO2. The epidermis was separated with tweezers, washed with IMDM 10% FCS and pushed through a 100 uM-pore nylon strainer (Falcon-BD biosciences, San Jose, CA) with the plunger of a 2 ml syringe to obtain a single-cell suspension. The cell suspensions were used for HLA-A2 typing by flowcytometric analysis as described below, by use of HLA-A2 specific monoclonal antibodies (mAb); used clones were BB7.2 and MA 2.1 from the American Tissue Culture Collection (ATCC, Rockville, MD).

### Flow cytometry

Phenotypic analyses were performed by flow cytometry. Cells were washed in PBS supplemented with 1% BSA and 0.02% NaN_3_ (PBA) and incubated for 30 min. at room temperature in the presence of appropriate dilutions in PBA of FITC, PE, PerCP or APC fluorochrome-conjugated specific mAbs to CD3, CD4, CD11c, CD14, CD25, CD1a, B7–H1 (i.e. Programmed Cell Death-Ligand 1, PD–L1), DC-SIGN, CD80, CCR7, CD163 (BD, San Jose, CA), BDCA3 (Miltenyi, Bergisch Gladbach, Germany), E-cadherin, Langerin, or CD83 (Beckman Coulter Immunotech), FoxP3 (eBioscience, San Diego, CA) or corresponding isotype-matched control mAbs (BD, San Jose, CA). For intracellular staining of Langerin, E-Cadherin and DC-SIGN, surface markers CD14, CD1a and CD11c were first stained as described above. Subsequently, cells were fixed and permeabilized using the BD Fix-Perm kit, following manufacturer's guidelines. Intracellular staining with PE-labeled DC-SIGN, Langerin, E-Cadherin or control IgG1 was performed for 30 minutes at 4°C in 1× permeabilization buffer. Cells were washed once with 1× permeabilization buffer and once with FACS buffer before analysis. The cells were subsequently analyzed, using a FACSCalibur and Cellquest-Pro FACS analysis software (BD, San Jose, CA).

### IL-10 and IL-12p70 release

DC migrated from human skin were harvested at the indicated time points and analyzed for functional IL-12p70 and IL-10 release as described [22]. Briefly, 1×10^4^ DC were incubated with 1×10^4^ J558-CD40L cells in the presence of 1000 U rhIFNγ/ml (Sanquin Blood Supply, Amsterdam, The Netherlands) in 100 µl IMDM with 10% FCS. After 24 h, the supernatants were collected and stored at −20°C until further analysis. IL-12p70 and IL-10 concentrations were determined by capture ELISA as previously described [23].

### Th and Treg differentiation assays

40,000 skin-emigrated DC were incubated with 0.5 µg/ml anti-CD3 (OKT-3, eBioscience, San Diego, CA) in 200 µl complete medium (i.e. IMDM supplemented with 10% fetal bovine serum (FBS; HyClone), 100 IU/ml sodium penicillin, 100 µg/ml streptomycin sulfate, 2 mM L-glutamine, and 0.01 mM 2-ME for 15 minutes at 4°C. After incubation, the DC were co-cultured with 20,000 CD4^+^CD25^−^ T cells (isolated by magnetic bead separation using the untouched CD4 isolation kit and anti-CD25 beads from Miltenyi, Bergisch Gladbach, Germany, according to the manufacturer's instructions) for 14 days. At day 7, 10 U/ml IL-2 (Strathmann Biotec, Hamburg, Germany) was added to the cultures. On day 14, T cells were harvested and analyzed by flowcytometry for CD3, CD4, CD25 and FoxP3 expression as previously described [24] or 50,000–100,000 T cells were stimulated with 0.5 µg/ml anti-CD3 (OKT-3) and 0.5 µg/ml anti-CD28 (clone 15E8) overnight. Supernatants were collected and were analyzed for cytokines secreted by the Th cells using a Th1/Th2/Th17 CBA kit (BD Biosciences, San Jose, CA) and an IL-22 ELISA kit (R&D systems, Minnesota, USA), following the manufacturer's instructions. At that time stimulated Th cells were harvested and RNA isolated to also assess transcript levels of Th1-, Th2-, Th17-, Th22-, or Treg-associated transcription factors and cytokines. In an alternative protocol 10,000 migrated and OKT3-loaded DC were cultured for 7 days with 1×10^5^ peripheral blood lymphocytes and analyzed by flowcytometry for CD3, CD4, CD25 and FoxP3 expression. In addition, at that time the cells were incubated for 4 hours with 50 ng/ml PMA and 500 ng/ml ionomycin in the presence of 0.5 µl/ml brefeldin A (GolgiPlug, BD Biosciences), stained for CD3 and CD4, and for FoxP3 and IFNγ (BD, San Jose, CA) after permeabilisation using the eBioscience Treg staining kit and subsequently analyzed, using a FACSCalibur and Cellquest-Pro FACS analysis software (BD, San Jose, CA).

### RNA Isolation and cDNA Synthesis

Total RNA was isolated using the RNeasy Plus Micro kit (Qiagen, Hilden, Germany). Contaminating genomic DNA was removed by using the gDNA Eliminator spin colums from the kit. The concentration and purity of the RNA was analyzed using the NanoDrop ND-1000 (Thermo Scientific, Wilmington, DE 19810 USA). cDNA was synthesized using oligo(dT)_20_ primers and the SuperScript III First-Strand Synthesis System for RT-PCR (Invitrogen, Carlsbad CA) according to the manufacturer's instructions. Input of RNA was 240 ng. After cDNA synthesis nuclease-free water was added up to a final volume of 80 µl.

### Real-time qRT-PCR

Transcripts were quantified by real-time quantitative polymerase chain reaction (qPCR) using an ABIPRISM 7900 Sequence Detector and pre-designed TaqMan Gene Expression Assays and reagents according to manufacturer's instructions (Applied Biosystems, Foster City, CA), as described previously [24]. Probes with the following Applied Biosystems assay identification numbers were also used: TBX21 (Tbet), Hs00203436_m1; GATA3, Hs00231122_m1; FOXP3, Hs00203958_m1; RORC1-2, Hs00172858_m1; RORC10-11, Hs01076112_m1, IL17A, Hs00174383_m1; human HPRT1 Endogenous Control (4333768T; Applied Biosystems) served as reference housekeeping gene. We validated all primers according to protocol. Mean relative mRNA expression was calculated using Pfaffl method [25].

### CD8+ T cell induction

To determine the capacity of skin explant-emigrated DC to prime specific CD8^+^ T cells, 2-day migrated DC were loaded with 1 µg/ml Mart-1_26–35L_ peptide (ALGIGILTV, obtained from LUMC, Leiden The Netherlands) in the presence of 3 µg/ml β_2_-microglobulin (Sigma-Aldrich) for 4–5 h at room temperature. Multiple bulk cultures of 1×10^6^ HLA-A2-matched CD8β^+^ T cell precursors were subsequently cultured with 5×10^4^ MART-1_26L–35_ peptide-loaded allogeneic skin-emigrated DC and with 1×10^6^ irradiated (80 Gy) CD8β–depleted autologous PBMC in Yssels medium supplemented with 1% human AB serum (ICN Biochemicals), 10 ng/ml IL-6, and 10 ng/ml IL-12 (R&D systems Inc,) in a 24-well tissue-culture plate. At day 1, 10 ng/ml IL-10 was added. After 10 days, CD8^+^ T cell cultures were analyzed for the presence of MART-1_26L–35_-specific CD8^+^ T cells by tetramer (Tm) staining as described previously [26]; cultures with Tm percentages exceeding 0.1% were considered positive. At this time, CD8^+^ T cells were restimulated with 1*10E5 JY cells, loaded with 10 ng/ml MART-1_26L−35_ peptide, and cultured for an additional seven days in the presence of 5 ng/ml IL-7 (Strathmann Biotec). After this additional round of restimulation, Tm positive cultures were pooled per experimental condition and the functional avidity of the Mart-1 primed CD8^+^ T cells was determined.

### CD8+ T cell functional avidity analysis

CD8^+^ T cells were cultured with titered peptide-loaded JY cells at an effector: target ratio of 2∶1 in 96-well round-bottom plate in the presence of 0.5 µl of GolgiPlug (BD Biosciences). After 5 h, cells were harvested, washed, and stained with allophycocyanin (APC)-labeled tetramer, and PE-labeled antiCD8 mAb. After fixation with Cytofix/Cytoperm solution and permeabilization with Perm/Wash solution (both from BD Biosciences), cells were labeled with FITC-conjugated anti-IFNγ mAb (BD Biosciences). Stained cells were analyzed on a FACSCalibur with Cellquest-Pro software (Becton Dickinson, San Jose, CA). Functional avidity was expressed as peptide concentration at which half maximal percentage of IFNγ-expressing CD8^+^ T cells was detected, as described previously [26].

### Statistical analysis

DC subset frequencies and marker expression levels, cytokine release levels, transcript levels, and specific T cell frequencies were compared between conditions using either the (paired) T test or the (repeated measures) one-way ANOVA or Friedman test with respective post-hoc Tukey or Dunn's multiple comparison analyses. Fractions of tetramer positive cultures were compared by the Fisher's Exact test and Pearson correlations between transcript levels of T cell transcription factors and cytokines were calculated. Prism 4.0 statistical software (GraphPad Software Inc., La Jolla, CA) was used. Differences and correlations were considered significant when *P*<0.05 in two-sided analyses.

## Results

### Predominant CD14^+^ DC migration from IL-10-conditioned or breast cancer-associated skin

**Figure 1:**
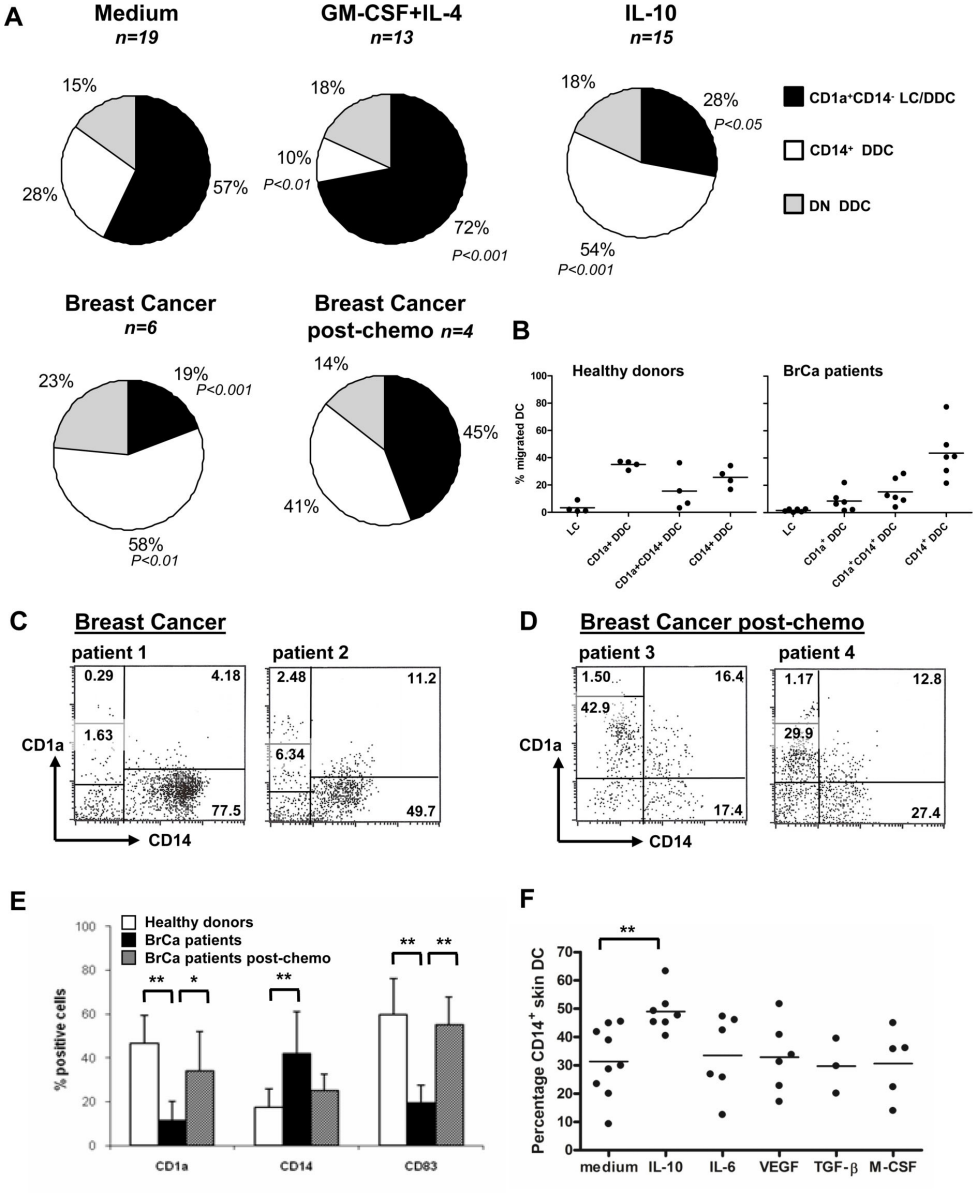
Dermal cytokine conditioning modulates subset distribution among skin-emigrated DC. **A**) Pie charts present the distribution of 2-day skin-emigrated CD1a^+^, CD14^+^ (including CD1a^+^CD14^+^) and double negative (DN) DC subsets for the indicated test conditions. Breast cancer explants were taken from tumor-overlying skin from chemonaive patients undergoing mastectomy. Post-chemo skin explants were similarly obtained from neoadjuvant chemotherapy-treated breast cancer patients. **B**) Distribution of Langerhans cells (LC) or CD1a^+^ and/or CD14^+^ dermal DC (DDC) among DC migrated from healthy donor explants or from explants taken from skin overlying breast tumors. Representative CD1a/CD14 FACS analyses of DC migrated from **C**) breast cancer patients and **D**) from breast cancer patients treated with neoadjuvant chemotherapy (percentages of the different subsets are indicated). **D**) Frequencies of CD1a/CD14/CD83 positive DC among the indicated test groups. Indicated statistical significance levels were relative to medium controls in repeated measures 1-way ANOVA (cytokine experiments) or unpaired Student T tests (breast cancer experiments). **E**) Known tumor-associated suppressive cytokines were i.d. injected and migrated DC subsequently harvested from skin explant cultures 2 days later; n = 3–9. CD14^+^ DC frequencies were significantly upregulated by IL-10 only (P<0.01, denoted by asterisks).

We previously reported that i.d. cytokine levels influenced the subset composition of migratory DC from human skin, with DC-stimulatory cytokines (GM-CSF and IL-4) inducing predominant migration of CD1a^+^ LC and/or DDC with a mature phenotype, and IL-10 inducing predominant migration of CD14^+^ DDC with an immature phenotype [19]. Here we confirm these findings (see Fig. 1A, n = 13–19) and additionally show that 48 h-migrated DC from skin explants taken from breast tumor-overlying skin, showed similar low frequencies of mature CD1a^+^ migratory DC and high frequencies of immature CD14^+^ subsets as observed subsequent to IL-10 conditioning (n = 6, Fig. 1A, 1B). CD1a^+^, CD1a^+^CD14^+^, and CD14^+^ DDC are subsets that range from more to less mature respectively [19]; we found a clear shift to a predominance of immature CD14^+^ subsets among DC migrated from breast tumor-overlying skin (Fig. 1C). Importantly, the migrated DC subset distribution appeared to be normalized subsequent to neo-adjuvant chemotherapy, confirming a relation to tumor load of the observed aberrant subset distribution (n = 4, Fig. 1A; for information on tumor response we refer to the Materials and Methods section). Representative CD1a vs CD14 FACS dot plots from breast cancer patients with and without neoadjuvant chemotherapy, illustrating the shifting DC subset distributions, are shown in Fig. 1C and 1D, respectively. Accompanying the tumor-associated shifts in the CD1a/CD14 frequencies, CD83 frequencies also shifted in concordance with a mature state of the CD1a^+^ DC and an immature state of the CD14^+^ DC (Fig. 1E). While it proved technically unfeasible to determine the responsible factor for the observed CD14^+^ subset skewing in cancer-conditioned explants (due to the limited size of mastectomy-derived skin samples), testing of a panel of six well-known cancer-associated suppressive cytokines (as well as PGE2, data not shown) clearly pointed to a unique role for IL-10 in this respect (see Fig. 1F).

### DC migrating from human skin comprise at least six phenotypically distinct subsets

**Figure 2:**
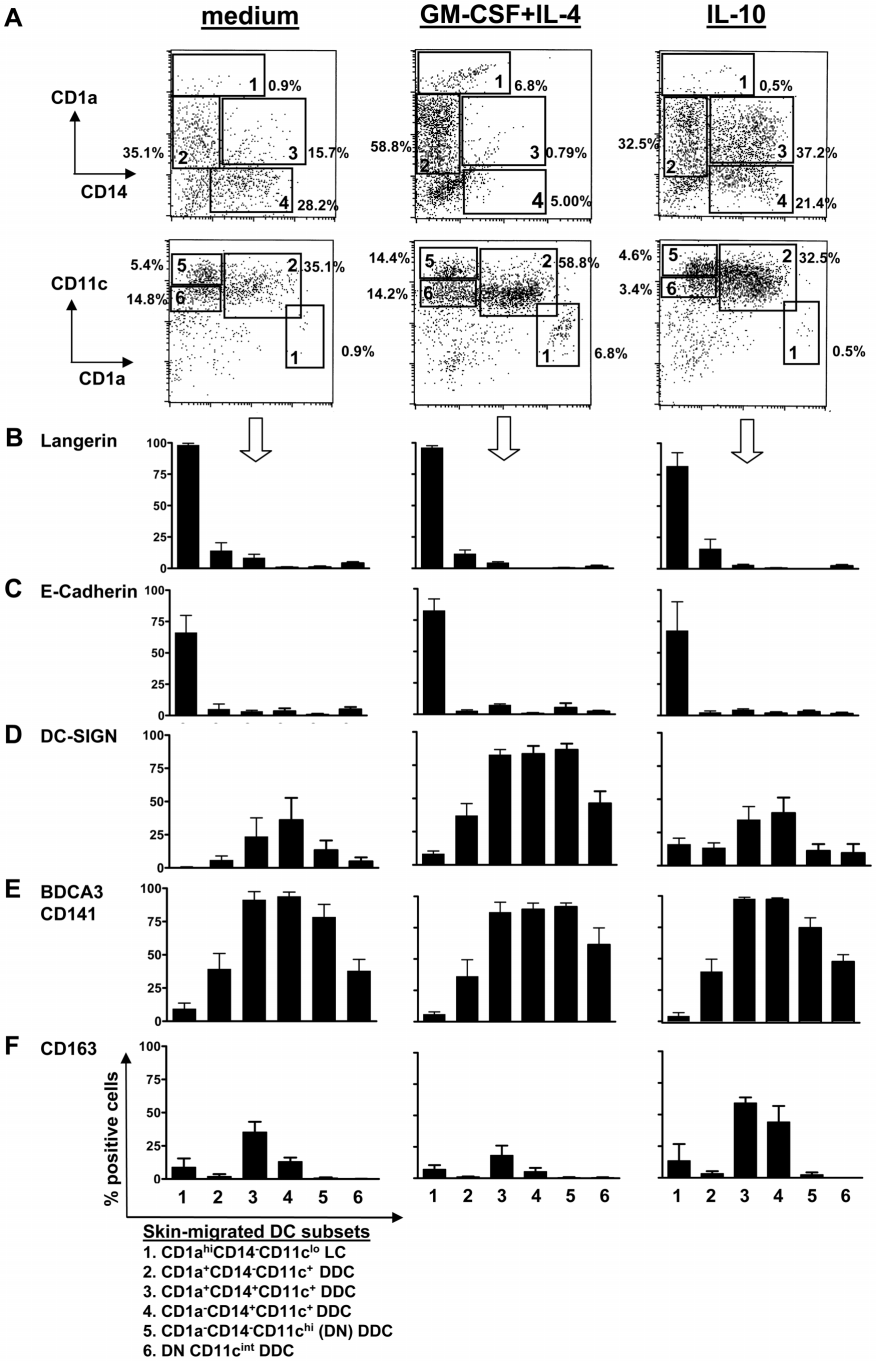
Human skin explant-emigrated DC consist of at least six subsets. **A**) DC, migrated from 2-day cultured skin explants, were gated by high Forward and Side Scatter properties and by differential CD1a, CD14 and CD11c expression levels (displayed by dot plots) to distinguish six separate subsets as listed at the bottom of the figure. Prior to culture, explants were i.d. injected with plain medium, GM-CSF+IL-4, or IL-10 as indicated. Percentages of the DC subsets corresponding to the depicted gates are shown. **B–F**) Expression levels of the indicated markers (in % positive cells, mean±s.e.m.) are shown within each of the DC subsets and for all three test conditions; n = 4.

We next set out to further delineate the various migratory skin DC subsets and how dermal cytokine conditioning affected their phenotype and migration rates. Based on CD1a, CD14, and CD11c expression (Fig. 2A) as well as subset-specific markers (Fig. 2B–F), six different populations or subsets were identified among DC that had migrated from 48 h-cultured full-thickness human skin explants. As shown in Fig. 2B and 2C, near-exclusive expression of intracellular Langerin and E-Cadherin on the small CD1a^hi^ population (with notably lower CD11c expression levels than the other subsets –Fig. 2A) clearly identified it as epidermis-derived LC (subset 1). Of note, Langerin expression was also observed on a small subpopulation of the more frequent CD1a^+^ DDC (subset 2). Intracellular DC-SIGN expression (up-regulated by i.d. administration of GM-CSF+IL-4) was observed in all five DDC subsets, but reached highest frequencies in the CD14^+^ subsets (subsets 3 and 4) and the CD1a^−^CD14^−^ (double negative, DN) subset with high CD11c expression levels (subset 5) (see Fig. 2A and 2D). Of note, these subsets also displayed the highest expression levels of the C-type Lectin BDCA3/CD141 (a.k.a. Thrombomodulin, Fig. 2E), a marker recently linked to DC subsets with particularly powerful cross-priming abilities [27], [28]. CD163, a macrophage marker associated with the M2 regulatory phenotype [29], was selectively expressed on the CD14^+^ subsets and up- and down-regulated by i.d. conditioning with IL-10 or GM-CSF+IL-4, respectively (see Fig. 2F).

### Cytokine conditioning affects migration rates of skin DC subsets and overall immune stimulatory versus inhibitory DC phenotype

**Figure 3:**
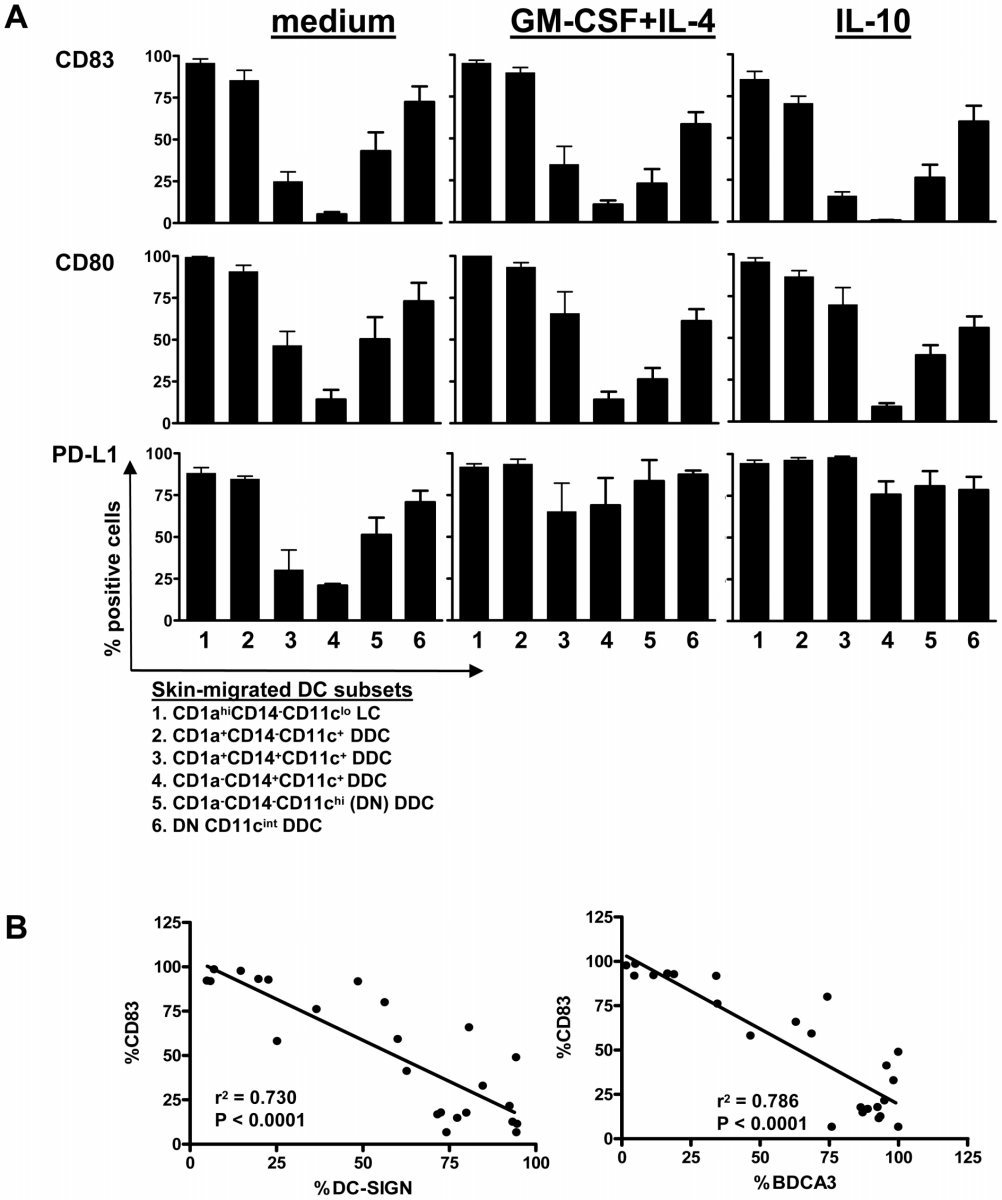
Human skin-emigrated DC subsets display different maturation states. **A**) DC subsets (designated 1–6, phenotypic definitions listed), migrated from 2-day cultured skin explants, display varying levels of the maturation-associated markers CD83, CD80, and PD-L1 n = 4 (in % positive cells, mean±s.e.m.). **B**) Significant reverse correlations between subset expression levels of DC-SIGN and BDCA3/CD141 on the one hand and CD83 on the other, demonstrate DC-SIGN and BDCA3 to be valid markers for immature skin-derived DC subsets under steady-state conditions (i.e. migrated from serum-free medium injected explants); n = 4.

**Figure 4:**
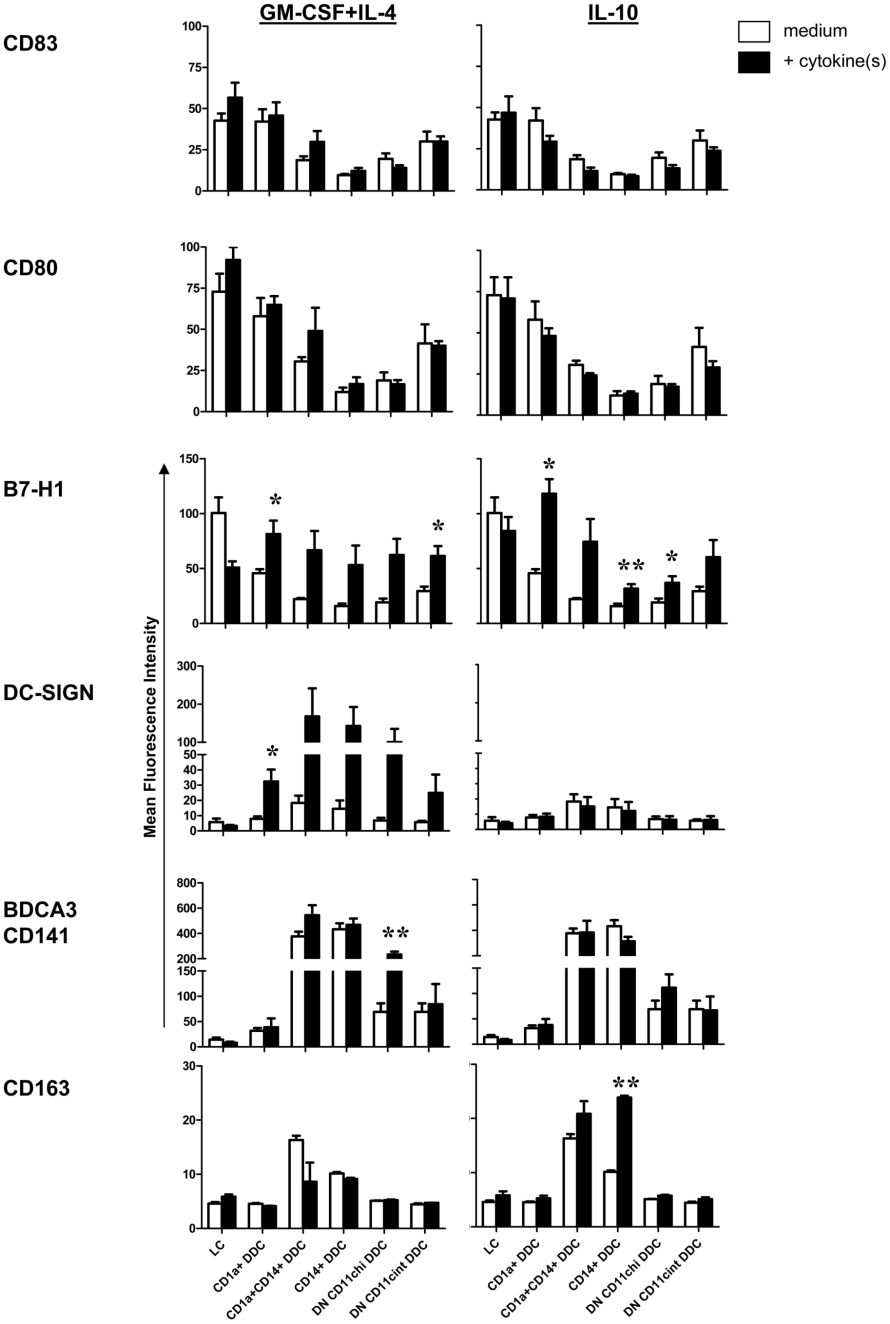
Expression (fluorescence intensity) levels of co-stimulatory, co-inhibitory or subset-defining markers on skin-emigrated DC subsets upon stimulatory or inhibitory cytokine modulation. DC subsets (phenotypic definitions listed), migrated from 2-day cultured skin explants i.d. injected with either medium (open bars) or GM-CSF+IL-4 or IL-10 (closed bars), display varying levels of the maturation-associated markers CD83, CD80, and PD-L1 or the subset defining markers DC-SIGN, BDCA3 and CD163; n = 4 (in mean fluorescence intensity [MFI], mean±s.e.m; NB: isotype control fluorescence levels of all subsets never exceed MFI of 5). *P<0.05, **P<0.01 by paired t-test, medium versus cytokine conditions.

The various DC subsets displayed different states of maturation as evidenced by differential expression levels of maturation and co-stimulatory markers (see Fig. 3A). Based on the expression of CD83, LC and CD1a^+^ DDC (subsets 1 and 2) clearly displayed a superior maturation state over the other subsets. Conversely, the CD14^+^ DDC subsets and the DN CD11c^hi^ DDC subset (subsets 3–5) were the least mature. Similar expression patterns were observed for CD80 (Fig. 3A) and CD40 and CD86 (not shown). Remarkably, the over-all maturation state of the various subsets, as judged by the maturation–associated markers CD83 and CD80, was hardly affected by the i.d. delivery of either the stimulatory cytokine cocktail GM-CSF+IL-4 or the inhibitory cytokine IL-10, both in terms of percentage positive cells and in mean fluorescence intensity, see Fig. 3A and Fig. 4 respectively. Interestingly, a highly significant reverse correlation between CD83 expression and expression of either DC-SIGN or BDCA3 on all six DC subsets migrated under steady state (i.e. medium control) conditions (Fig. 3B) identified the latter two as reliable markers for an immature state of migratory skin DC. In contrast to co-stimulatory markers, the co-inhibitory molecule PD-L1 (B7–H1) was up-regulated on all DDC subsets by both GM-CSF+IL-4 and IL-10 (see Fig. 3A and 4). Remarkably, this was not the case for LC; indeed upon i.d. delivery of GM-CSF+IL-4 migratory LC significantly down-regulated PD-L1 levels (Fig. 4). In addition, intensity levels of intracellularly stained DC-SIGN were strongly up-regulated on all migrated DDC subsets upon dermal GM-CSF+IL-4 conditioning. High CD163 levels that were further up-regulated by IL-10, were detected specifically on both CD14^+^ DDC subsets, consistent with a regulatory macrophage-like phenotype (Fig. 4).

**Figure 5:**
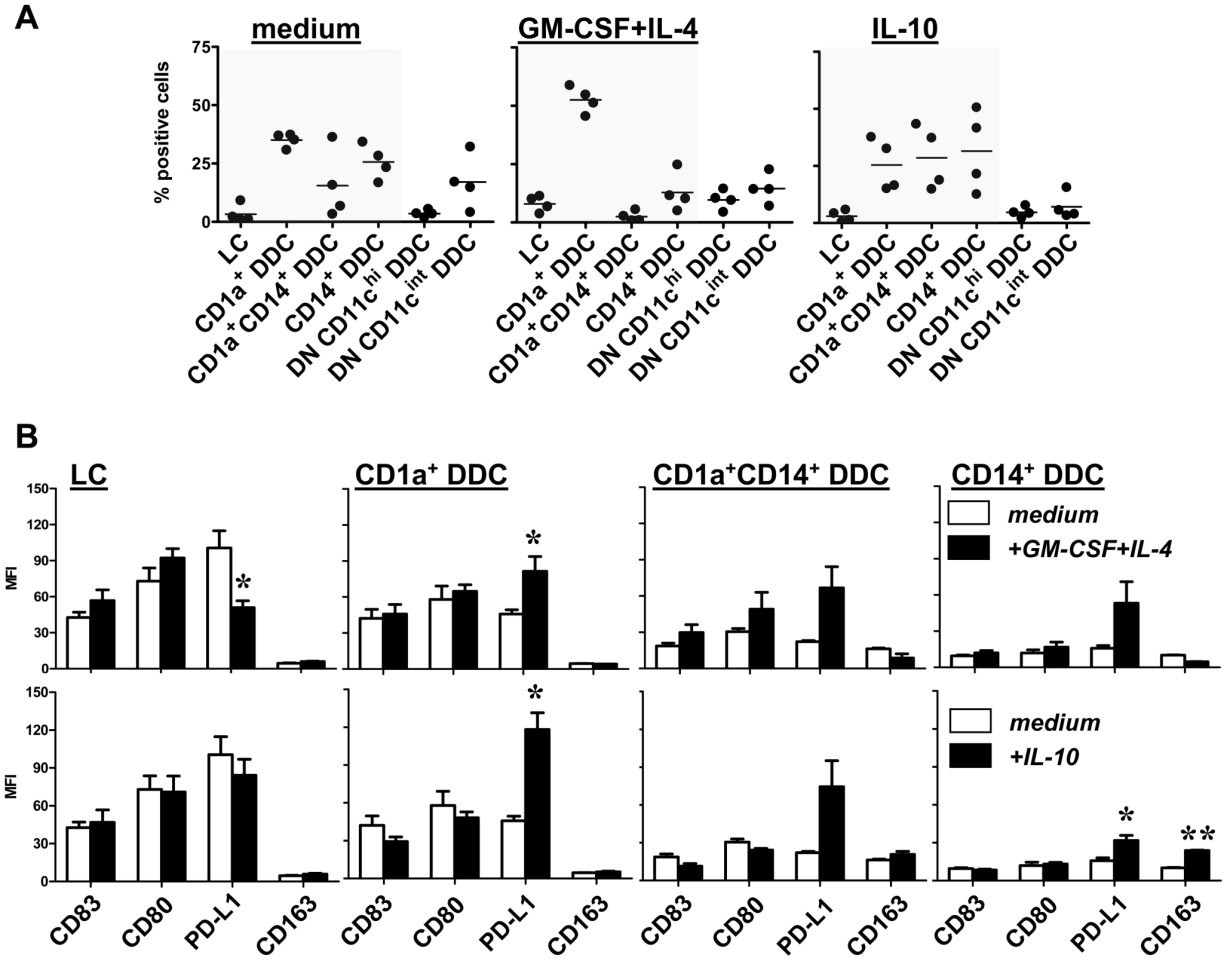
T cell-stimulatory or –inhibitory phenotypic features of the CD1a^+^ and/or CD14^+^ skin-emigrated LC and DDC subsets and their relative distribution upon cytokine modulation. **A**) DC subset frequencies among 2-day skin-emigrated DC from skin explants i.d. injected with medium, GM-CSF+IL-4 or IL-10, prior to migration; n = 4 (mean±s.e.m.). High-lighted areas contain results for the CD1a and/or CD14 positive LC and DC subsets shown in (**B**). **B**) Side-by-side comparison of the mean fluorescence intensity levels of the co-stimulatory markers CD83 and CD80 and the inhibitory marker PD-L1 and the M2 regulatory macrophage-related marker CD163 per LC and DDC subset with and without cytokine modulation; n = 4, *P<0.05, **P<0.01 by paired t-test, medium versus cytokine conditions.

While i.d. delivery of cytokines did not alter the maturation state of the individual DC subsets -as judged on the basis of CD83 and CD80 expression, it did alter their relative contribution to the total migrated DC population (Fig. 5A). As compared to medium controls, i.d. conditioning by GM-CSF+IL-4 resulted in increased frequencies of the more mature LC and CD1a^+^ DDC subsets, while i.d. conditioning by IL-10 led to increased frequencies of the immature CD1a^+^CD14^+^ and CD14^+^ DDC subsets (see high-lighted areas in Fig. 5A). In Fig. 5B cytokine-modulated levels of the co-stimulatory markers CD83 and CD80 are shown side by side with corresponding levels of the co-inhibitory molecule PD-L1 and the regulatory M2-associated macrophage marker CD163 for each of the individual LC and CD1a and/or CD14^+^ DDC subsets. The combination of consistently low levels of co-stimulatory markers (CD83 and CD80) and up-regulated PD-L1 and CD163 levels most likely signify a T cell-suppressive make-up of the CD14^+^ DDC subsets, in particular upon i.d. IL-10 modulation. The preponderance of the CD14^+^ DDC subsets among skin-emigrated DC subsequent to i.d. IL-10 delivery would thus predict poor overall T cell activation. In contrast, high levels of co-stimulatory levels on the LC and CD1a^+^ DDC subsets might ensure T cell activation, despite up-regulated PD-L1 levels upon cytokine modulation. To ascertain the actual effects of the observed cytokine-induced shifts in DC subset composition and phenotype on subsequently induced T cell responses, we next investigated the ability of the skin-emigrated DC to support and skew Th cell differentiation and to prime and expand specific CD8^+^ effector T cells.

### Modulation of IL-10 and IL-12p70 release

**Figure 6:**
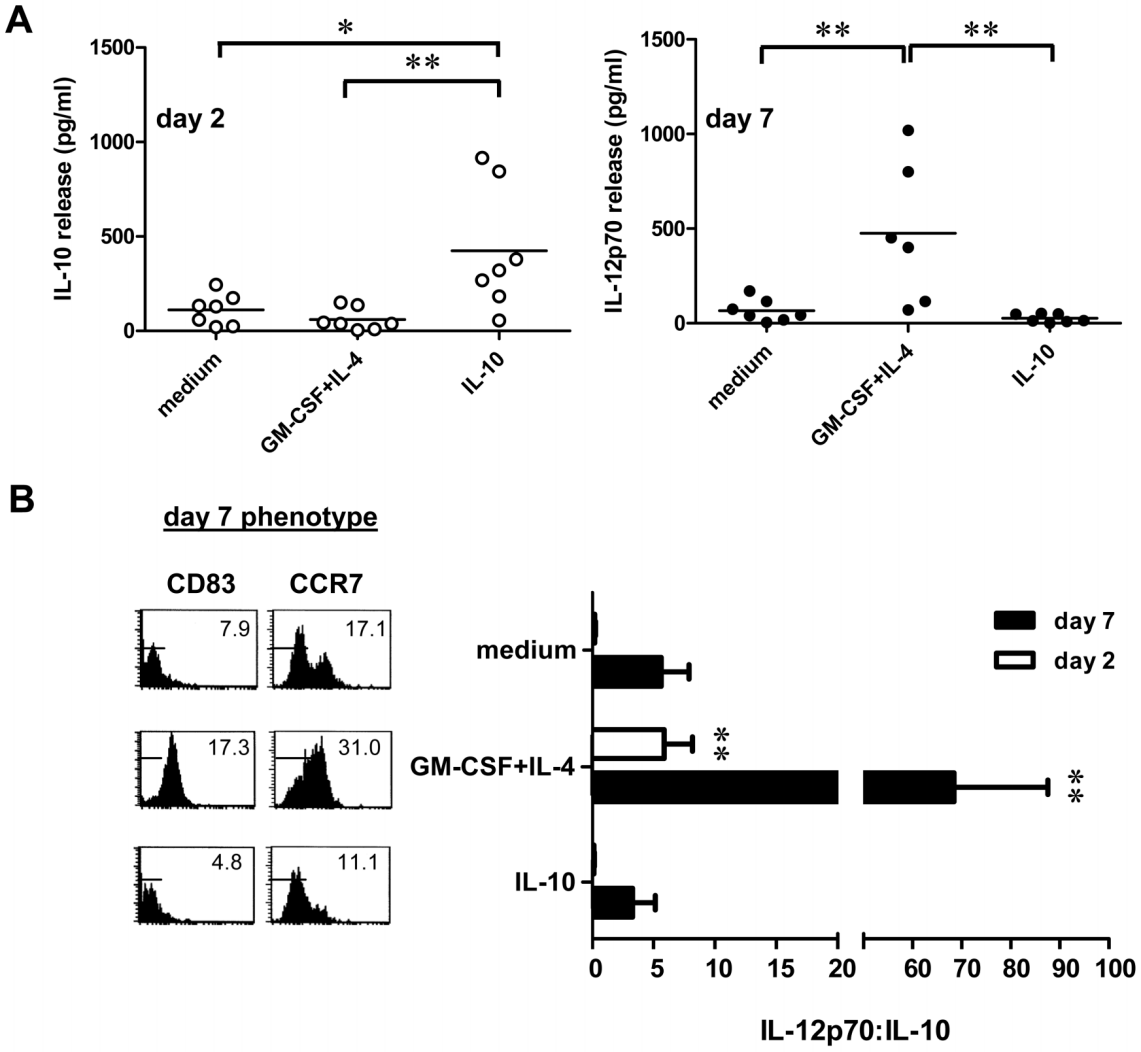
Dermal cytokine conditioning modulates IL-10 and IL-12p70 release by skin-emigrated DC. Skin explants were i.d. injected with medium or the indicated cytokines and cultured for 2 days at which time explants were discarded. Migrated cells were harvested at day 2 or 7, thoroughly washed, and stimulated by CD40L-expressing J558 cells in the presence of IFNγ (1000 IU/ml). Supernatants were harvested 24 hr later and IL-10 and IL-12p70 concentrations determined by ELISA. **A**) Cytokine release is shown in pg/ml per 10,000 cells per 24 h for the indicated conditions and time points. Means ±s.e.m. from 6–7 experiments are shown. **B**) Mean IL-12p70 and IL-10 concentrations were divided to obtain IL-12∶IL-10 ratios for the indicated cytokine conditions and time points (N = 6–7). The obtained ratios are displayed in relation to CD83 and CCR7 expression on the skin explant-emigrated DC at day 7 after the start of explant culture (mean fluorescence intensities are listed and corresponding isotype control levels marked). Asterisks denote significant differences (*P<0.05, **P<0.01).

As an indication of their ability to support durable T cell differentiation, migrated (and thoroughly washed) DC were tested for CD40L-stimulated release of IL-12p70 and IL-10 at two and seven days after start of migration (Fig. 6A). High levels of IL-10 (around 400 pg/ml) were only released by DC emigrated from IL-10-conditioned explants and exclusively in early stages of migration (day 2). Lower levels of IL-10 production (≤100 pg/ml) were observed for the other test conditions at this time (Fig. 6A). IL-10 release in any of the tested conditions had dropped below the detection limit of the employed ELISA by day 4 (data not shown). Of note, IL-12p70 release followed reverse kinetics with highest levels at day 7, which were significantly elevated upon i.d. delivery of GM-CSF+IL-4 (see Fig. 5A). Lower, but still elevated, levels for the latter condition were detected at day 2 (mean 125 pg/ml (range 15–305) vs. 6.5 pg/ml (range 0–29) for medium controls, *P*<0.05) and 7.0 pg/ml (range 0–27) in the IL-10 condition. In order for the observed IL-12p70 release to result in proper type-1 T cell activation, the skin-emigrated DC need to preserve their mature T cell-stimulatory phenotype as well as their ability to home to the paracortical T cell areas of the lymph node (mediated by the CCR7 chemokine receptor). We therefore related expression of CD83 and CCR7 at day 7 to the IL-12∶IL-10 ratio. As shown in Fig. 6B, significantly higher IL-12∶IL-10 release ratios favorable to immune activation were observed at day 2 and 7 for DC migrated from explants conditioned by GM-CSF+IL-4 (predominantly CD1a^+^), and were related to a preserved expression at day 7 of CD83 and CCR7. In contrast, DC migrated from IL-10-conditioned skin (predominantly CD14^+^) displayed low IL-12∶IL-10 ratios and expressed neither CD83 nor CCR7.

### Cytokine-mediated conditioning of the dermis: effects on Th cell differentiation

**Figure 7:**
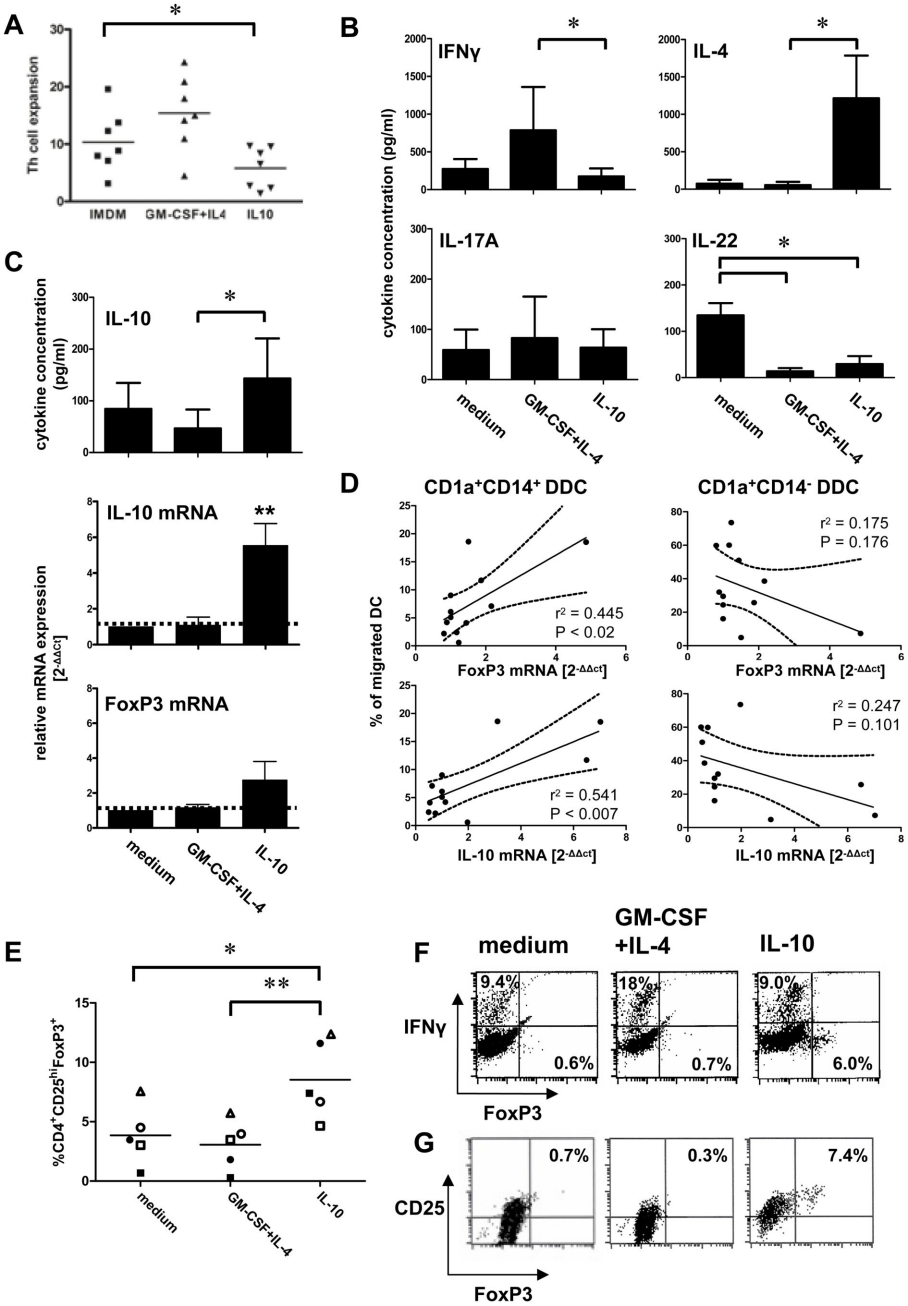
Effects of dermal cytokine conditioning on the induction of Th cell differentiation by skin-emigrated DC. Two days after injection of medium with or without cytokines, skin-emigrated DC were harvested, pulsed with anti-CD3 and co-cultured for 2 weeks with CD4^+^CD25^−^ allogeneic T cells. **A**) Th cell expansion factors after 2 weeks of co-culture. **B**) Cytokine release levels (measured after 24 h stimulation with anti-CD3/anti-CD28, n = 4; mean±s.e.m.) and **C**) IL-10 release and relative IL-10 and FoxP3 mRNA expression levels (n = 3, mean±s.e.m) of the primed Th cells. Significant differences in appropriate paired statistical tests were determined versus medium controls (unless otherwise indicated) and denoted by asterisks: *P<0.05, **P<0.01. **D**) Correlation between CD1a^+^CD14^+^ and CD1a^+^CD14^−^ DDC frequencies among skin-emigrated DC (for all tested conditions, n = 3) and FoxP3 and IL-10 transcript levels (statistical analysis by Pearson correlation). **E**) Superior expansion and/or induction of CD25^hi^FoxP3^+^ Tregs by DC migrated from IL-10 conditioned skin explants; open symbols: skin-emigrated DC from three donors were co-cultured with unsorted allogeneic lymphocytes; closed symbols: skin-emigrated DC from two donors were co-cultured with sorted CD4^+^CD25^−^ T cells. *P<0.05, **P<0.01 in a repeated measures ANOVA with Tukey post-hoc test. **F**) FoxP3 versus IFNγ expression in allogeneic CD4^+^CD3^+^ T cells (both by intracellular staining upon PMA/ionomycin stimulation for 4 hours), primed by DC migrated from conditioned human skin explants and **G**) flowcytometric Treg analysis in 14-day co-cultures of migrated skin DC with allogeneic CD4^+^CD25^−^ T cells; test conditions and percentages of FoxP3^+^ or IFNγ^+^ CD4^+^ T cells or of CD25^hi^FoxP3^+^ Tregs are indicated.

We next studied the ability of IL-10-modulated skin DC to skew differentiation of allogeneic Th cells. To this end migrated DC were pre-loaded with anti-CD3 and cultured with CD25^−^ Th cells over a period of 14 days. Next, the Th cells were restimulated with anti-CD3 and anti-CD28 for 24 h and supernatants and mRNA were subsequently obtained to determine cytokine release and Th cytokine and transcription factor transcript levels. DC that had migrated from IL-10 conditioned explants induced poor Th cell expansion rates (Fig. 7A) with an apparent Th2 switch and decreased release of IL-22 (Fig. 7B). Moreover, they induced significant increases in IL-10 release and transcription, concomitant with increased FoxP3 mRNA expression levels (Fig. 7C). Indeed, IL-10 and FoxP3 transcript levels were significantly correlated (r^2^ 0.748, P<0.006, data not shown). These data are consistent with the induction of potentially suppressive Tr1-like cells by CD14^+^ DDC emigrated from IL-10 conditioned skin. This notion was further supported by a significant correlation between both FoxP3 and IL-10 transcript levels and frequencies of migrated CD1a^+^CD14^+^ DDC, i.e. subset 3, which also expressed CD141 (Fig. 2, Fig. 4) and the PD-L1 expression levels and migration rate of which were both notably increased by IL-10 modulation (Fig. 5A,B). In contrast, an inverse trend was observed for CD1a^+^ DDC that lacked CD14 expression and displayed high co-stimulatory levels, see Fig. 7D. Of note, in 7-day co-cultures of skin emigrated and anti-CD3 loaded DC with unsorted allogeneic peripheral blood lymphocytes, highest frequencies of CD25^hi^FoxP3^+^ Tregs were observed in cultures with DC emigrated from IL-10 conditioned skin explants (Fig. 7E, n = 3 open symbols). Whereas intracellular IFNγ expression upon activation with PMA and ionomycin was observed in FoxP3^−^ Th cells in these cultures, this was not the case for the CD25^hi^FoxP3^+^ cells (Fig. 7F), providing further functional evidence of their Tr1/Treg identity. To confirm the actual induction/conversion of Tr1/Tregs from conventional CD4^+^ T cells, cells from 14-day co-cultures with sorted allogeneic CD25^−^ Th cells were also stained for CD25 and intracellular FoxP3 expression. As demonstrated by Fig. 7G, CD25^hi^FoxP3^+^ Tr1/Tregs were induced only by DC emigrated from IL-10 conditioned skin (n = 2, closed symbols in Fig. 7E).

### Cytokine-mediated conditioning of the dermis: effects on CD8^+^ effector T cell priming

**Figure 8:**
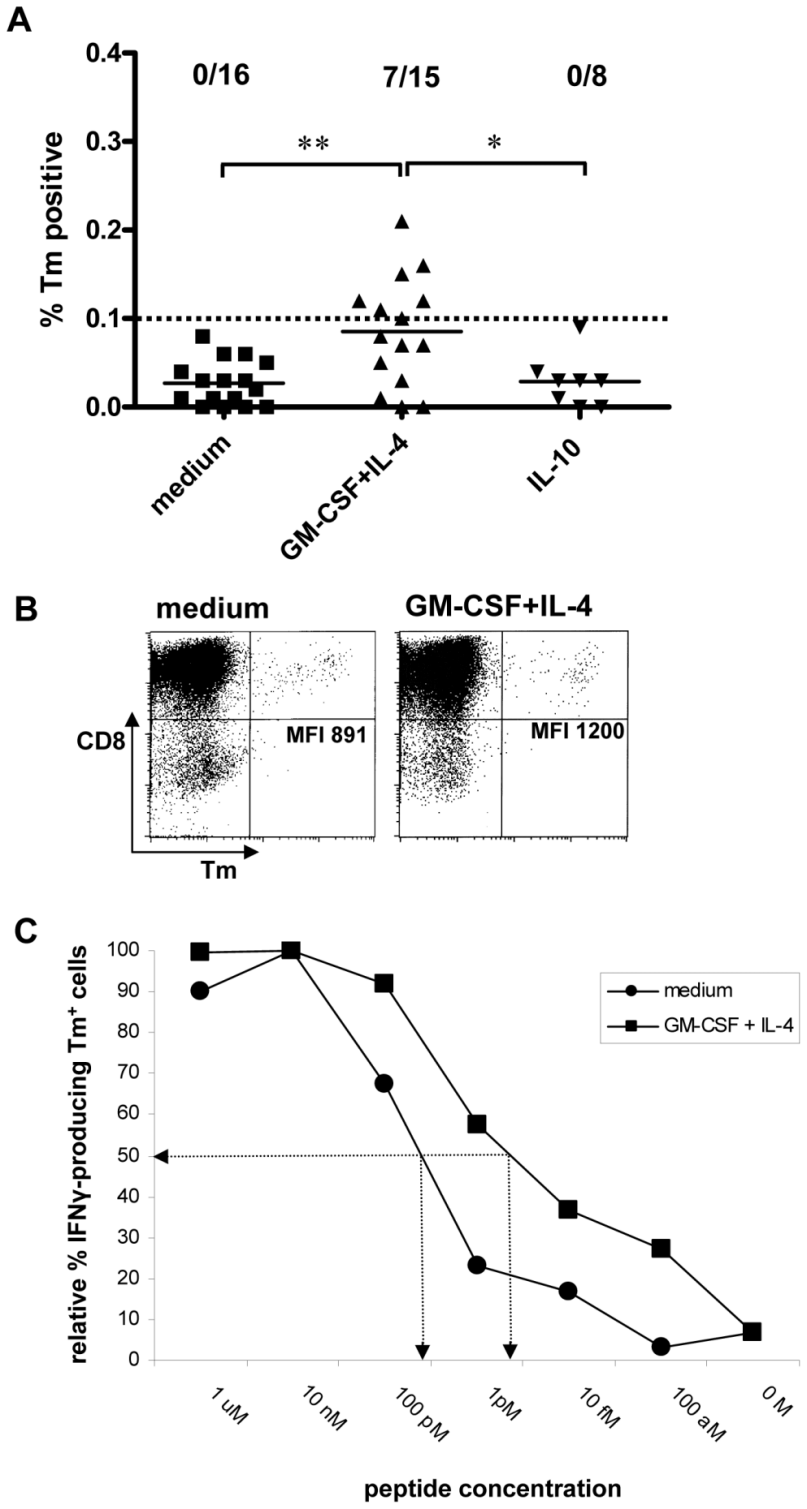
Efficient priming of high-avidity CD8^+^ T effector cell by skin-emigrated DC upon dermal conditioning with GM-CSF+IL-4. A) Multiple parallel bulk cultures were set up of HLA-A2 matched allogeneic CD8β^+^ T cells from three different donors together with Mart-1_26L−35_ peptide loaded DC, emigrated from skin explants that were i.d. injected by medium or cytokines as indicated. Ten days later Mart-1_26L−35_−specific T cell frequencies were determined in all bulk cultures by HLA-tetramer (Tm) binding. Aggregate results from all 3 experiments are shown. *P<0.05, **P<0.01 in Fisher's Exact test. B) Seven days after restimulation with Mart-1_26L−35_ peptide loaded JY cells, Tm positive cultures were pooled. Sufficient numbers were obtained from one T cell donor to undertake functional avidity analyses for the medium and GM-CSF+IL-4 conditions. Tm binding levels in these two bulk cultures are shown (MFI: Mean Fluorescence Intensity). Note: no Tm positive cultures were obtained for any of the IL-10 conditions. C) Stimulation of the pooled bulk cultures with JY cells, loaded with titrated amounts of the Mart-1_26L−35_ peptide, followed by intracellular IFNγ expression read-out, showed CD8^+^ effector T cells from the GM-CSF+IL-4 condition to be of higher avidity than those from the medium condition (half-maximal frequencies of IFNγ producing Tm^+^ T cells for both test conditions and the corresponding peptide concentrations used for JY loading are indicated by arrows).

To assess the effects of cytokine-modulated DC subset migration on CD8^+^ effector T cell priming, migrated DC from HLA-A2^+^ explants were loaded with the immune-dominant Mart-1-derived epitope Mart-1_aa26L−35_ and cultured with HLA-A2-matched allogeneic CD8β^+^ T cells. Multiple parallel bulk cultures per condition (n = 3–6) were set up with DC from three different skin donors. After 10 days specific expansion of Mart-1_aa26L−35_ reactive CD8^+^ T cells was checked by HLA-tetramer staining. Combined data from the three priming experiments are shown in Fig. 8A. After this one round of stimulation, tetramer positive cultures were exclusively found after stimulation by DC from GM-CSF+IL-4-conditioned skin for all 3 donors tested (1/4, 4/6, and 2/5 positive cultures, respectively). In one donor massive T cell death in the IL-10-modulated DC cultures precluded reliable tetramer analysis. T cells were subsequently re-stimulated with peptide-loaded JY cells and seven days later re-analyzed for tetramer binding (Fig. 8B). Even then no tetramer positive cultures were observed for the IL-10 conditions, whereas in one donor a T cell bulk primed by medium control DC did bind tetramers, albeit at a low intensity, suggestive of low T cell receptor binding affinity. This was borne out by functional avidity analysis, showing half-maximal IFNγ release by these tetramer positive T cells at approximately 100-fold higher peptide concentrations than T cells primed by DC from GM-CSF+IL-4-conditioned skin (see Fig. 8C).

## Discussion

Most of our knowledge of the functionality of skin DC subsets has been gleaned from mouse studies. In human skin, functional studies have been hampered by low DC numbers and by the inability to manipulate them in a targeted fashion, as has been achieved in mice through conditional knock-out of specific DC subsets [30]. Much of what we do know of human skin subsets has been the result of research on the dermal delivery of cancer vaccines, in which new avenues have been explored to specifically target cutaneous DC subsets with the ability to efficiently prime Th1 and CTL responses [31]. Tumor-induced systemic suppression of DC development and maturation may well interfere with these functions. We previously showed the effects of the cancer-associated suppressive cytokine IL-10 on the phenotype and allogeneic T cell priming ability of human skin-emigrated DC [19]. In the current study we more extensively profiled various DDC subsets and studied the effects of cancer-related and cytokine-mediated conditioning of the human dermis on the subset composition and phenotype of the skin-emigrating DC population in relation to the induction and skewing of CD4^+^ and CD8^+^ T cell differentiation. Although the numbers of migrated cells were too low to allow for sorting of the different subsets, our findings show dramatic shifts in the relative migration rates of the DC subsets with clear impact on the induction of both functional Th and CD8^+^ T cells.

In keeping with findings by others (reviewed by Teunissen *et al.* [32]), intracellular staining for Langerin and E-Cadherin positively identified the CD1a^hi^ subset among the human skin explant-emigrated DC as LC, while intracellular expression of DC-SIGN identified the five remaining subsets as DDC (either CD1a^+^ and/or CD14^+^, or DN). DC-SIGN was upregulated by i.d. administration of GM-CSF and IL-4 in all five DDC subsets. This up-regulation was most likely dependent on IL-4, previously shown to be primarily responsible for the induction of DC-SIGN [33]. Interestingly, cytokine conditioning mostly affected the migratory balance between the CD1a^+^ and CD14^+^ skin DC subsets, more so than their general maturation state, and apparently left the migration rates of both DN DDC subsets relatively unaffected. Importantly, the CD14^+^ DDC subsets were phenotypically immature, characterized by high levels of BDCA3/CD141, a marker recently associated with DC subsets with cross-presentation abilities [27], and their relative migration rates were increased by dermal IL-10 conditioning. In a comparative analysis with CD14^−^ DDC, CD14^+^ DDC were previously shown to be poor inducers of allogeneic T cells and to require high DC∶T cell ratios for Th1 induction [14]. Here we show that dermal conditioning by IL-10 increased their PD-L1 expression levels, while maintaining low levels of co-stimulatory molecules, and led to high IL-10 release levels upon CD40L-mediated stimulation. This resulted in Th2 and Tr1/Treg induction, accompanied by IL-10 transcription and release upon co-culture with CD4^+^CD25^−^ T cells, and an inability to prime CD8^+^ effector T cells. In keeping with these observations, Banchereau *et al.* have recently shown that IL-10 released by CD14^+^ DDC into the immune synaps is mainly responsible for their inability to prime functional CTL [8], while interference with Immunoglobulin-like transcript inhibitory receptors (ILT) inhibits their type-2 cytokine skewing ability [34].

Of note, our previous [19] and current findings, identifying CD14^+^BDCA3/CD141^+^ DC as an immature migratory subset with immune regulatory properties, are in line with a recent report by Chu et al. showing IL-10 release and functional Treg induction by the same subset with suppressive effects on skin inflammation [18], suggesting a role in the maintenance of homeostatic tolerance. In addition we now show an increased frequency of the CD14^+^ DDC subset among DC migrating from IL-10- and cancer-conditioned skin. Of particular interest in this context is the IL-10-induced upregulation of the M2-macrophage associated marker CD163 on CD14^+^ migratory DDC. This marker was previously reported on skin-resident macrophages [35], which were also found to express DC-SIGN. Although DC-SIGN and CD163 are both expressed by the CD14^+^ DDC, which, moreover, functionally and morphologically resemble macrophages [19], we have nevertheless designated them DC, because 1) they are migratory unlike dermal macrophages which are generally regarded as sessile [36], 2) they express BDCA1/CD1c [19], a typical feature of DDC [35] and 3) because they can develop from migrated CD1a^+^ DC through a CD1a^+^CD14^+^ intermediate (designated DDC subset 3 in this study –see Fig. 1 and 2) [19]. The latter observation raises an important issue that as yet remains unresolved: are the observed sub-populations among the migrated human DDC genuine subsets, or rather representations of the same DDC in various states of (trans-)differentiation? Indeed, we have previously found compelling evidence, both from cytokine-conditioned and irritant-treated skin explants, that LC and CD1a^+^ DDC can trans-differentiate during migration to the CD14^+^ macrophage-like DDC subset in an IL-10-dependent fashion [19], [37]. Conversely, Larregina *et al.* reported the differentiation of LC from dermal CD14^+^ precursors [38]. Finally, it has even been suggested that an inflammatory environment in the dermis may lead to the trans-differentiation of sessile macrophages to migratory (CD14^+^) DDC, possibly accounting for the observed reduced densities of macrophages in human skin explants upon culture [31], [38]. Altogether these studies paint a picture of an inter-related population of dermal DC and macrophages in flux, trans-differentiating into each other, as dictated by environmental conditions. Indeed, the IL-10-conditioned CD14^+^ DDC with a DC-SIGN^+^BDCA3^+^CD163^+^ phenotype are highly reminiscent of both tumor-associated macrophages and DC, adopting similar M2-like immunosuppressive traits [39]–[41]. As a model for cancer-induced suppression of DC in the skin microenvironment, we also studied the migration of DC from explants derived from skin overlying breast tumors, for which a reduced LC density was previously reported [42]. Similarly to the IL-10-modulated skin explants, a remarkable shift to immature CD14^+^ DDC migration was observed in these skin explants, taken from mastectomy specimens from chemo-naive patients. The small size of the skin specimens obtained from these patients precluded neutralization experiments to identify the responsible suppressive factor. However, testing a panel of tumor-associated culprits pointed to IL-10 as the most likely candidate, since it was the only tested cytokine that could affect the balance of migrating CD1a^+^ and CD14^+^ DDC. This is in keeping with other reports demonstrating the unique ability of IL-10 among other suppressive factors to modulate the maturation and migration of fully differentiated DC [40], [43], [44]. Importantly, reduction of tumor load by neoadjuvant chemotherapy led to normalization of the migratory DDC subset distribution, providing evidence for tumor involvement in the observed aberrant migration of predominantly CD14^+^ DDC.

In contrast to IL-10, i.d. delivery of GM-CSF+IL-4 effected predominant migration of CD1a^+^ DDC and LC and resulted in Th1 induction (evidenced by increased IFNγ release levels). In addition, GM-CSF+IL-4-modulated DDC and LC were superior inducers of anti-melanoma CD8^+^ effector T cells, which were shown to exhibit increased functional avidity. Klechevsky *et al.* first reported a functional dichotomy between human LC and CD14^+^ DDC with the former preferentially activating CTL and the latter B cell responses [45]. Our own genome-wide transcriptional profiling studies of LC versus CD1a^+^ DDC, clearly showed the latter to be more activated under steady state conditions [46]. In keeping with findings by others, our studies show that LC only make up a small minority of the “crawl-out” DC derived from cultured full-thickness skin explants (typically between 1–5%) [32]. While i.d. delivery of GM-CSF and IL-4 prior to culture increased this frequency to about 10%, their relative under-representation in the tested conditions leaves the exact contribution of LC to the observed effects on T cell induction in doubt. Nevertheless, our current findings that IL-10-induced increases in CD14^+^ DDC migration correspond to increased Th2/Treg skewing and that GM-CSF+IL-4-induced increases in LC and CD1a^+^ DDC migration correspond to efficient and high-avidity CD8^+^ T cell priming, are in line with the observations made by Klechevsky *et al* [8], [45].

Whereas default Th1 induction by skin DC was previously shown to depend on IL-23 rather than IL-12p70 release [14], we found evidence for a GM-CSF and IL-4-induced release of IL-12p70 which most likely accounts for the increased induction and expansion of Th1 and high-avidity CD8^+^ effector T cells. Several reports have indicated that *in vitro* generated LC as well as primary LC and DDC, isolated from human skin, are poor IL-12p70 producers [7], [9], [47]. Our data indicate that LC and/or DDC are capable of high-level IL-12p70 release upon intracutaneous activation by GM-CSF and IL-4, but only at late time points following migration, i.e. after 5 to 7 days. This is in sharp contrast to previous findings for *in vitro* cultured MoDC, which reach a so-called “exhausted” state 48 h post-stimulation, resulting in an inability to release IL-12p70 and to induce Th1 cells and CTL [48], [49]. Preferred early IL-12p70 release is in keeping with the proposed acute inflammatory role of MoDC *in vivo* [50]. In contrast, skin-resident DC, with a very low turn-over and migration rate [51], predominantly release IL-10 under steady state conditions. Only in the presence of strong maturational signals such as the pro-inflammatory cytokines GM-CSF and IL-4, which induce a mature phenotype that can be sustained for up to 7 days following the initiation of migration, do skin-emigrated DC release high levels of IL-12p70 in response to CD40L stimulation, and then contribute to the induction of type-1 T cell-mediated immunity. IL-10/IL-12p70 release kinetics thus indicate a powerful default tolerance maintained by migrating skin DC that can only be lifted by strong activating signals resulting in durable DC maturation.

Finally, and remarkably, i.d. delivery of either IL-10 or GM-CSF/IL-4 resulted in down-regulated transcription and release of IL-22. In view of the respective inhibitory and stimulatory effects of IL-10 and GM-CSF+IL-4 on type-1 T cell activation, this observation is in keeping with the recognized role of IL-22 in skin homeostasis and inflammation as well as its apparent minor role in cell-mediated immunity against viruses and/or cancer [52].

In conclusion, IL-10 conditioning of human skin leads to predominant migration of immature CD14^+^BDCA3/CD141^+^ DDC subsets, which display a suppressive M2-like phenotype, i.e. low co-stimulatory marker levels combined with up-regulated PD-L1 and CD163, and dominant IL-10 release, and which do not support CD8^+^ effector T cell expansion, but rather induce type-2 and IL-10 producing Th cells as well as CD25^hi^FoxP3^+^ Tregs. In contrast, GM-CSF+IL-4 conditioning leads to predominant migration of CD1a^+^ LC and DDC subsets in a sustained mature state that predominantly release IL-12p70 and can induce both Th1 and tumor-specific CD8^+^ high-avidity effector T cells. Our data suggest overriding immune suppressive effects of IL-10 in tumor-conditioned skin and argue in favor of the incorporation of GM-CSF and IL-4 in adjuvants for transcutaneously or intradermally delivered cancer vaccines.
